# Oxidative stress: An essential factor in the process of arteriovenous fistula failure

**DOI:** 10.3389/fcvm.2022.984472

**Published:** 2022-08-11

**Authors:** Ke Hu, Yi Guo, Yuxuan Li, Chanjun Lu, Chuanqi Cai, Shunchang Zhou, Zunxiang Ke, Yiqing Li, Weici Wang

**Affiliations:** ^1^Department of Vascular Surgery, Union Hospital, Tongji Medical College, Huazhong University of Science and Technology, Wuhan, China; ^2^Clinic Center of Human Gene Research, Union Hospital, Tongji Medical College, Huazhong University of Science and Technology, Wuhan, China; ^3^Center of Experimental Animals, Huazhong University of Science and Technology, Wuhan, China; ^4^Department of Emergency Surgery, Union Hospital, Tongji Medical College, Huazhong University of Science and Technology, Wuhan, China

**Keywords:** arteriovenous fistula, AVF failure, oxidative stress, antioxidant, remodeling

## Abstract

For more than half a century, arteriovenous fistula (AVFs) has been recognized as a lifeline for patients requiring hemodialysis (HD). With its higher long-term patency rate and lower probability of complications, AVF is strongly recommended by guidelines in different areas as the first choice for vascular access for HD patients, and its proportion of application is gradually increasing. Despite technological improvements and advances in the standards of postoperative care, many deficiencies are still encountered in the use of AVF related to its high incidence of failure due to unsuccessful maturation to adequately support HD and the development of neointimal hyperplasia (NIH), which narrows the AVF lumen. AVF failure is linked to the activation and migration of vascular cells and the remodeling of the extracellular matrix, where complex interactions between cytokines, adhesion molecules, and inflammatory mediators lead to poor adaptive remodeling. Oxidative stress also plays a vital role in AVF failure, and a growing amount of data suggest a link between AVF failure and oxidative stress. In this review, we summarize the present understanding of the pathophysiology of AVF failure. Furthermore, we focus on the relation between oxidative stress and AVF dysfunction. Finally, we discuss potential therapies for addressing AVF failure based on targeting oxidative stress.

## Introduction

For over half a century, arteriovenous fistula (AVFs) has been recognized as a lifeline for patients requiring hemodialysis (HD) (1, 2). Globally, more than 2.6 million patients suffered from end-stage renal disease (ESRD) in 2010, and this number is expected to increase to 5.4 million over the next 20 years (3, 4). With its higher long-term patency rates and lower probability of complications, such as infection and thrombosis, as well as lower long-term mortality in comparison to other types of access, AVF is strongly recommended by guidelines, with wide consensus from experts in different areas as the first choice of vascular access for HD patients, and its proportion of application is gradually increasing. Furthermore, due to its safety and reliability, AVF is also recommended for use in the pediatric population (5).

**FIGURE 1:**
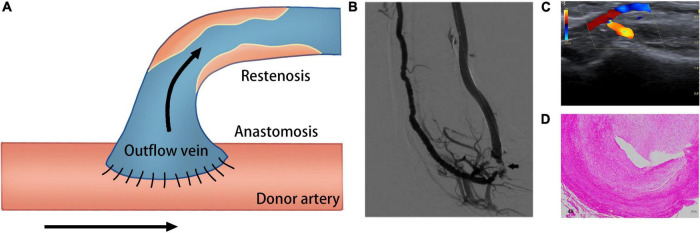
Schematic of creation of the AVF. **(A)** Creation of AVF with end-to-side technique. **(B)** Venography of the AVF; arrow shows the occlusion of the arterial inflow (233). **(C)** Duplex ultrasound image shows fistula occlusion of the arterial inflow (234). **(D)** Hematoxylin and eosin staining shows neointima formation in the venous outflow tract of AVF (235).

Despite technological improvements and advances in the standards of postoperative care, many deficiencies are still encountered in the use of AVF related to its high incidence of unsuccessful maturation and stenosis. It was reported that in about 60% of cases, successful dialysis use is not established within half a year following AVF creation (6). AVF failure is characterized by a tight perianastomotic stenosis, which leads to a narrower lumen and lower blood flow (Figure 1). AVF stenosis is the consequence of uncontrolled aggressive neointimal hyperplasia (NIH) combined with failure of early outward remodeling and subsequent excessive inward remodeling (7). Many studies have demonstrated that several vascular biology pathways may be responsible for this pathological change, including inflammation, oxidative stress, hypoxia, and alterations in hemodynamic shear stress (8–10).

There is substantial evidence that oxidative stress has novel roles throughout the entire process of AVF maturation, including preoperative underlying mechanisms, intraoperative surgical injury, and postoperative hemodynamic changes (11, 12). Oxidative stress is defined as dysregulation between the production of reactive oxygen species (ROS) and the endogenous antioxidant defense mechanisms, the so-called “redox state.” When present at relatively low concentrations, ROS tend to maintain stability in the intracellular environment through the role of second messengers. However, redundant ROS disrupt the redox balance, which subsequently leads to DNA and protein damage, lipid peroxidation, and irreversible cell damage and death (13).

In this review, we summarize present understanding of the pathophysiology of AVF failure. Furthermore, we focus on the relationship between oxidative stress and AVF dysfunction. Finally, we discuss potential therapies for addressing AVF failure based on targeting oxidative stress.

## Biology of arteriovenous fistula maturation

### Definition of maturation

Maturation is the vascular remodeling process that renders AVF suitable for being routinely prescribed for dialysis. This process should be monitored by an experienced vascular access team for postoperative complications during the first 2 weeks. Further investigation is required by 4–6 weeks to estimate whether AVF maturing as expected (14).

However, there are currently no satisfactory clinical criteria for defining AVF maturation. The arm-raising test can reveal larger collateral veins or accessory veins earlier, which are indicative of AVF maturation failure and stenosis. In addition, more objective follow-up examinations should be performed regularly, such as ultrasound Doppler examinations and radiological examinations. The value of shear stress and venous diameter can be used to accurately predic functional changes after AVF creation (15–17). According to the blood vessel diameter and hemodynamic parameters, minimum standards for satisfactory arteriovenous fistula maturation within 4 weeks have been established (vessel diameter > 4 mm and blood flow > 400 ml/min) (18–21).

### Arteriovenous fistula maturation and outward remodeling

Following the AVF creation process, the moderate adaptation of the structure and function occurring near the anastomosis area are essential for AVF maturation. Firstly, a low-resistance circuit constructed by the inflow artery and outflow vein through direct anastomosis or shunting immediately triggers an increase in blood flow and violent changes in wall shear stress (WSS), which remains relatively stable for a certain period of time (22–24). The substantial increase in the WSS leads to the initial venous lumen expansion by mechanical stretching.

In addition to the short-term outward remodeling caused by the direct change in blood flow pressure, the activation of endothelial cells (ECs) caused by the significant increase in WSS is also considered to play a vital role in the early remodeling process (25). Ideally, the large amount of nitric oxide (NO) produced by the activation of ECs simultaneously expands the inflow artery and outflow vein, inhibits the development of NIH and gradually restores WSS to the baseline level. Furthermore, the excessively released NO not only directly enhances the vasodilation effect but, through combination with free radicals, is also converted to peroxynitrite, which stimulates matrix metalloproteinases (MMP?2 and MMP?9) (26–28). Appropriate upregulation matrix metalloproteinases trigger extracellular matrix (ECM) degradation and decompose the vascular middle layer elastic fibers, resulting in sustained luminal expansion (28–30). In summary, the abovementioned multiple biological mechanisms jointly facilitate the remodeling of venous outflow and preserve a stable level of WSS and luminal diameter at different periods for subsequent regular dialysis.

## Pathogenesis of arteriovenous fistula failure

### Definition of arteriovenous fistula failure

As the number of patients with ESRD increases, the demand for hemodialysis treatment is also growing. As the preferred choice of vascular access for hemodialysis, AVF failure remains a significant barrier to successful fistula use. While there are some criteria to define successful AVF maturation, the clinical definition of AVF failure is less clear due to confusion between the various types and stages of failure. The development of NIH or thrombosis near the fistula anastomosis is the major cause of lumen narrowing and AVF failure. NIH-induced failures include activation and migration of vascular cells and remodeling of the ECM, where the complex interplay of cytokines, adhesion molecules, and inflammatory mediators results in poor adaptive remodeling.

### Thrombosis

Hemodialysis access thrombosis is a multifactorial process that usually marks short-term lumen blockage and loss of function in the dialysis access. The German pathologist Rudolf Virchow proposed three elements of thrombosis: blood flow, blood vessel wall, and coagulation components. This is commonly referred to as the three elements of Virchow (31). Patients with ESRD are at risk of bleeding due to platelet dysfunction and anticoagulants use during hemodialysis. However, at the site of vascular access, dysfunctional platelets can precipitate clot formation (32–35).

End-stage renal disease patients also have an increased risk of thrombosis due to heightened systemic inflammatory responses. Due to abnormal systemic metabolism, the balance of prothrombotic and antithrombotic in the circulatory system of ESRD patients is disrupted, which may be an important factor for the increased risk of thrombosis in ESRD patients. Several prothrombotic mediators were significantly elevated in chronic kidney disease (CKD) patients. Elevation of fibrin and inactivation of prothrombin directly lead to a hypercoagulable state (36). Other soluble thrombosis-related proteins, including soluble tissue factor, von Willebrand factor, and C-reactive protein (CRP), are also involved in pro-coagulation. High levels of phospholipid antibodies (caused by uremia) and high levels of low-density lipoprotein can also exacerbate the hypercoagulable state (37, 38).

In addition to cytokine-induced hypercoagulability, systemic inflammatory states are further exacerbated by endothelial dysfunction due to coagulation abnormalities. One study showed that endothelial glycocalyx disruption was observed in ESRD patient specimens, and that this also contributed to an increased risk of thrombosis (39). Tissue factor is an important procoagulant that activates the extrinsic coagulation cascade, and uremic ECs also release small extracellular vesicles, called microparticles, loaded with tissue factor, which leads to increased thrombosis (40–42).

The dialysis process has also been shown to facilitate the activation of platelets to induce a hypercoagulable state. Semipermeable membranes used in a dialysis apparatus can activate platelets, and multiple studies have shown that dialysis can increase levels of circulatory p-selectin, von Willebrand Factor, and D-dimer (43–45). In the hypercoagulable state, platelet and leukocyte aggregation can induce atherosclerosis and thrombosis. In addition to the inflammatory environment of the circulatory system, repeated acupuncture injury during dialysis can also cause inflammation of the lumen local endothelium. Platelets can lead to enhanced adhesion through the release of inflammatory mediators such as monocyte chemoattractant protein 1 (MCP-1), vascular cell adhesion protein-1 (VCAM-1), intercellular adhesion molecule 1 (ICAM-1), interleukin-1β (IL-1β), and tumor necrosis factor α (TNF-α) and ultimately form plaques or thrombosis.

### Neointimal hyperplasia

As the most common scene, invasive intimal hyperplasia is a major cause of vascular access failure (46–48). Stenosis in HD occurs in the anastomotic appendages, especially on the venous side. The pathogenesis of AVF vascular access disorders is generally divided into upstream and downstream events. Upstream events include the previous uremia environment, hemodynamic changes caused by surgical trauma, oxidative stress, hypoxia, and other initial factors of vessel wall damage. Downstream events represent changes in different cells in response to upstream vascular injury, including endothelial disturbance, activation of different types of cells, and proliferation. The development of NIH is a complex process that requires endothelial activation, phenotypic changes, and migration of vascular and immune cells, and the proliferation of multiple cell types together leads to intimal hyperplasia. The cell types involved include lymphocytes as well as vascular smooth muscle cells (VSMCs) and fibroblasts. Local histology is characterized by SMA-positive, vimentin-positive myofibroblasts and secretory smooth muscle cells as well as a minority of contractile smooth muscle cells (49–51).

There are several different hypotheses about the origin of cells that cause NIH in AVF, including activation of adventitial and medial cells located in the venous *in situ* vessel wall, infiltration and migration of cells from adjacent arteries, and differentiation of inflammatory cells transported locally from the circulatory system. Different experimental AVF animal models have been used to identify clear sources of cells that promote neointima formation and have provided support for these hypotheses. Using a porcine arteriovenous graft model, Misra and colleagues initially demonstrated that the cells that underwent early proliferation following arteriovenous graft implantation, which led to venous stenosis, had originated from the adventitia and media (52). Roy-Chaudhury and colleagues also demonstrated in an AVF model that venous tissue fibroblast differentiation contributes to the formation of venous stenosis (53). Cheng et al. successfully bred RFP-Stop *^flox/flox^*-GFP/Wnt1-Cre+ mice by crossing Wnt1-Cre and RFP-Stop*^flox/flox^*-GFP transgenic mice (54), the biggest feature of which is AVF surgery. In the crossed mice, anterior carotid smooth muscle cells but not jugular smooth muscle cells were labeled with green fluorescent protein (GFP). The authors found that after AVF creation, nearly 50% of the intimal proliferative cells showed GFP positivity by immunofluorescence staining, confirming that the GFP-positive cells were derived from smooth muscle cells of the inflow tract artery. The role of cells from circulating blood has also been explored in recent years. Castier et al. demonstrated that myeloid cells do not promote NIH in AVF arteries (55). Misra et al. demonstrated that monocyte involvement contributes to AVF remodeling and stenosis through a monocyte depletion experiment (3).

## Molecular mechanism of arteriovenous fistula failure

**FIGURE 2:**
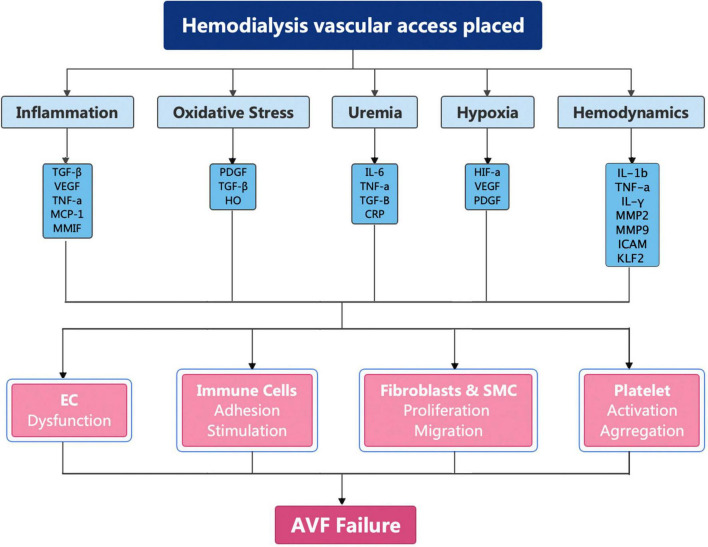
Molecular mechanism of AVF failure.

Arteriovenous fistula have been recommended as the first choice for HD by the National Kidney Foundation Kidney Disease Outcomes Quality Initiative on account of the superior long-term patency rates, fewer complications, and lower financial burden (14, 56–58). However, less than 60% of AVFs will be functional after 12 months (59–61). The pathogenesis of AVF failure is caused by inflammation, uremia, hypoxia, and shear stress changes (62–65). These factors cause upregulation of cytokines, which broadly activate fibroblasts, smooth muscle cells, immune cells, and platelets (Figure 2).

### Inflammation

Inflammation occurs throughout the whole life cycle of AVFs, including in the form of systemic inflammatory homeostasis disorder in patients with ESRD as well as local inflammatory responses resulting from surgical trauma at the time of AVF establishment.

The local inflammatory response often manifests as increased infiltration of local macrophages (CD68) and lymphocytes (CD3), which are especially more prevalent in uremic patients (66). These changes are evidenced by upregulation in many inflammatory cytokines, such as IL-6, IL-8, MCP-1 and plasminogen activator inhibitor-1 (PAI-1), as well as some proliferate cytokines, such as transformative growth factor-β (TGF-β) (67–69). Studies have shown that IL-6 and TNF-α play important roles in promoting early thrombosis in AVF, and increases in CRP and fibrinogen also lead to AVF failure (70, 71). Evidence from both experimental AVF models and clinical samples demonstrate that the dominant reason for the increase in inflammatory mediators is due to the massive release of macrophage migration inhibitory factor, which drives inflammatory cells to move toward the neointima, which leads to vascular wall thickening (72–74). Macrophage migration inhibitory factor induces changes in the expression of downstream proteins by binding to CD74 receptor, chemokine receptor 2, and chemokine receptor 4 (73). Changes in secreted protein brought about by inflammatory cells result in upregulation of many cytokines, IL-8, MCP-1, and vascular endothelial growth factor A (VEGF-A) through extracellular signal regulation and the p38 mitogen-activated protein kinase pathway (72). As a potent chemokine, MCP-1 plays a vital role in a variety of vascular diseases by promoting the migration and proliferation of monocytes and macrophages, EC activation, and proliferation and phenotypic switching of smooth muscle cells (75–78). MCP-1 is increased in expression after mouse AVF creation and becomes a mediator of AVF dysfunction and failure. In the mice AVF model, MCP-1 knockout resulted in significantly less NIH and improved patency rates (79).

Tumor necrosis factor α is another important inflammatory cytokine in NIH (80). In addition to mediating early immune responses, VSMC and EC can also produce TNF-α through an autocrine pathway in response to hypoxia, ROS, and local inflammation accumulation. TNF-α promotes the production of IL-1β and prostaglandin E2. TNF-α can induce fibroblast proliferation and migration, and these changes are greatly enhanced by insulin-like growth factors (81). Although it has a pro-apoptotic effect, apoptosis can only occur when the nuclear factor kappa B (NF-κB) signaling pathway is inactivated. Therefore, TNF-α promotes the proliferation of smooth muscle and fibroblasts in the AVF neointima by releasing inflammatory factors rather than by causing apoptosis.

### Oxidative stress

After establishment, AVFs are often exposed to an environment of oxidative stress that involves multiple factors, including pre-existing systemic inflammation and intraoperative injury, and postoperative remodeling in ESRD patients. Multiple studies have demonstrated that markers of oxidative damage, including 8-hydroxy-2-deoxyguanosine and 4-hydroxy-2-nonenal, are upregulated in AVF outflow tract venous tissue (82). The increased synthesis and secretion of ROS and the depletion of antioxidant substances lead to the disruption of oxidative balance, which in turn stimulates many signaling pathways that regulate various processes, including the promotion of cell proliferation and migration, and ECM secretion (11, 83). Luo et al. study has shown that the expression of 4-hydroxynonenal increased after downregulation of glutathione *S*-transferase α4, while its overexpression reduced 4-hydroxynonenal levels and inhibited SMC proliferation in mice with CKD, playing a vital role in CKD-induced neointima formation and AVF failure (84). Sustained excess superoxide anion rapidly oxidatively inactivates NO, which further leads to the uncoupling of endothelial nitric oxide synthase (eNOS) to produce superoxide, which is the main cause of endothelial dysfunction due to oxidative stress (85). These stimuli further increase lumen narrowing by increasing levels of potential mitogens, including platelet-derived growth factor (PDGF), endothelin 1, and the proliferation-stimulating factor TGF-β (11).

Heme oxygenase (HO) is a rate-limiting enzyme that promotes the catabolism of heme. HO exerts antioxidant, anti-inflammatory, and anti-apoptotic functions in cells through its downstream products (86–89). Recent studies have shown that HO plays an important role in promoting extravascular remodeling and preventing NIH (89). The expression of the HO-1 gene was significantly upregulated in VSMCs after AVF surgery in mice, which may be a protective effect stimulated by oxidative stress. In HO-1 knockout mice, the outflow tract vessel wall becomes thinner and there are increases in lumen area, patency rate, and levels of the pro-inflammatory and pro-oxidative mediator MCP-1 (90). We explain the specific production pathways of oxidative stress and the corresponding important molecules and signaling pathways in more detail in the subsequent parts of this article.

### Uremia

Before the creation of AVF, the inherent uremia in ESRD patients increases inflammation and oxidative stress (91, 92). Many inflammatory cytokines, including IL-6, TNF-α, and TGF-β, are elevated in the serum of CKD patients. In addition, mineral metabolism disorder in the blood of patients with ESRD may also aggravate the vascular wall thickness. Uremia has also been shown to affect vascular homeostasis through such mechanisms as affecting the activity of cellular calcium channels, increasing fibrosis, increasing insulin resistance, and increasing vascular wall calcium phosphate deposition (93, 94). Vascular calcification may also affect AVF remodeling. In clinical studies, elevated serum levels of CRP and sclerostin, an osteogenic protein with increased expression specifically in calcified VSMCs, were found to be independent predictors of AVF failure (95). Other vascular calcification markers such as fetuin-A, osteopontin, and bone morphogenetic protein-7 were also associated with the development of AVF complications in a cohort of dialysis patients (96). However, a prospective clinical study came to the opposite conclusion (97). The study concluded that serum concentrations of mineral metabolites in patients were not substantially related to AVF failure. Since it is possible that the concentration of minerals in the circulatory system may not truly reflect the degree of cellular calcification, the relationship between calcification and AVF failure requires more advances in mechanism research to give us a more definitive answer.

Inflammation-induced impaired platelet function and endothelial wall dysfunction accelerate the development of atherosclerosis. These complex synergistic changes further contribute to the susceptibility to inward remodeling after AVF placement.

### Hypoxia

Apart from the sustained hypoxic background existing in the circulatory system of patients with previous CKD, hypoxia results from the damage to adventitia and the dissection of vasa vasorum vessels during the creation of AVF. Repeat needle sticks during cannulation for dialysis are also thought to exacerbate hypoxic injury. Sustained hypoxia promotes angiogenesis, inflammation, and proliferation, resulting in NIH.

Among the various upregulated genes, increased levels of hypoxia-inducible factor 1 α (HIF1α) have been confirmed, which is shown to mediate NIH in both animal models and human specimens (10, 98). Under normoxic conditions, HIF1α hydroxylated by proline hydroxylase undergoes ubiquitination by either the Von Hippel Lindau tumor suppressor or E3 ubiquitin ligase and is ultimately degraded (99). HIF1α expression is stabilized under hypoxic conditions and affects many downstream molecular mechanisms that lead to angiogenesis, inflammation, cell proliferation, and collagen deposition (100). VEGF is recognized as one of the most important downstream mediators of hypoxia. VEGF acts primarily by binding to two receptors, VEGFR-1 and VEGFR-2. VEGFR-1 promotes endothelial growth by activating tyrosine kinases. VEGFR-1 can also exacerbate the local inflammation levels in AVF via macrophage activation (101). As a tyrosine kinase receptor, VEGFR-2 acts primarily through the phospholipase-cγ protein kinase-c pathway. VEGFR-2 is more highly expressed in the circulatory system and mainly plays a role in promoting the proliferation and differentiation of ECs (101–103). VEGFR-2 can also promote VSMC proliferation through regulation of extracellular signals and Akt signaling (104). Recent studies have shown that downregulation of VEGF-A gene expression can reduce IH formation during the setting of AVF. The polymorphism of VEGF-A gene is also considered to be an important factor affecting the occurrence of AVF occlusion (105).

Apart from VEGF, PDGF is another important cytokine activated by hypoxia. The platelet-derived growth factor family includes PDGF and VEGF. Each growth factor can be produced by a variety of cells, and its receptors are all tyrosine kinase receptors. *In vivo* monocytes/macrophages are the main cells that synthesize PDGF. PDGF acts on phosphorylated platelet-derived growth factor β receptor to prompt fibroblasts and VSMCs to enter the cycle of division and proliferation. Recent studies have demonstrated the function of PDGF in regulating VSMC phenotype switching, vascular fibrosis, and NIH (106, 107).

### Hemodynamics

The increased magnitude of vessel WSS and the alteration of flow patterns are likely to be the primary events after AVF creation that promote AVF adaptation (108). In response to dramatic hemodynamic changes after AVF creation, adaptive remodeling is needed to return the WSS to a stable level. In response to the hemodynamic changes, the AVF will undergo adaptive remodeling to bring the WSS back to a stable level. However, the presence of vascular lesions and systemic abnormalities contributes to the failure of AVFs to mature due to post-maturation venous stenosis and/or impaired external remodeling. The influence of WSS on venous inward remodeling mainly comes from two aspects: one is the direct effect of WSS upregulation on adjacent cells, and the other is the influence of disturbed hemodynamic near the anastomosis.

The elevation of WSS upregulates local inflammatory factors such as Il-1β, TNF-α, and interferon-γ (IF-γ) (109, 110). In addition, WSS has been shown to induce negative cell remodeling proliferation through VEGF-A, MMP2, MM9, and ICAM-1. WSS activates vascular endothelial cadherin and platelet endothelial cell adhesion molecule-1 (PECAM-1) by altering the mechanical tension state of cells, further inducing cell shape changes that alter cell migration ability (111–113). Persistent WSS also affects vasodilation by affecting HO and reducing NO synthesis (114).

Normally, vessels experience unidirectional stable laminar flow, but the chaotic, disorderly oscillatory flow generated by the vessel wall near the AVF anastomosis leads to the activation of a series of downstream pathways. These pathways directly or indirectly induce ECs to selectively express atherogenic and thrombogenic genes (115).

Oscillating WSS interacts with mechanical pressure receptors via the EC surface, resulting in increased EC autocrine proliferative pathways, upregulation of mitogen-activated protein kinases, nuclear translocation of NF-κB, and downregulation of Kruppel-like factor 2 (KLF2) expression and induced EC proliferative, pro-inflammatory and pro-oxidative state transitions (116). The disturbance of blood flow can also stimulate VSMC migration and proliferation, accelerating NIH development (117, 118).

Use of an external support device, VasQ (fixed metal stent near the anastomosis), to create an AVF has been reported to improve fistula maturity and patency by reducing flow disturbance around the anastomosis (119).

## Sources of oxidative stress in arteriovenous fistula failure

### Definition of oxidative stress

Oxidative stress is stonewalled by the universal definition of an imbalance between oxidants and antioxidants. When subjected to various harmful stimuli, the body produces excessive ROS with the depletion of antioxidants. The accumulation of free radicals eventually leads to tissue damage and various diseases. ROS are a group of oxygen reduction products that can be generated by enzymatic and non-enzymatic systems and (120). Although oxidative stress routinely plays a novel role in the regulation of diverse cellular functions and biological processes, uncontrolled ROS may mediate varying degrees of tissue and cellular damage.

Nicotinamide adenine dinucleotide phosphate (NADPH) oxidase, mitochondrial enzymes, xanthine oxidase, lipoxygenase, myeloperoxidase, and unconjugated eNOS are major contributors of ROS generated in blood vessel systems. The ROS products mainly include superoxide anion (O_2_^–^), hydroxyl radical, and reactive nitrogen species (RNS) in the form of free radicals, and the non-radical form of hydrogen peroxide (H_2_O_2_). Oxygen-related reduction products often exist in the form of free radicals due to the presence of an unpaired electron. Because of their strong chemical reactivity, these substances often produce irreversible cell damage in addition to mediating biological behavior. Hydrogen peroxide, which is a stable two-electron reduction product and not a free radical, often acts as a small molecule second messenger to activate downstream signaling pathways. Numerous studies have shown that oxidative stress is a key pathophysiological pathway in the progression of atherosclerosis, lower extremity ischemia, and other cardiovascular diseases (121, 122). Specimens from the outflow tract veins of patients with AVF and basic research in recent years have confirmed that ROS is an important cause of AVF failure and neointima production.

### Reactive oxygen species mediated cellular signaling

This functional protein network exists under the regulation of redox signaling, resulting in subsequent changes in signal outputs, enzyme activity, gene transcription, and other processes. The main mechanism of ROS-mediated cellular signaling is achieved by efficiently oxidizing thiolals in target proteins (123). It’s reported that about 20% of all thiolals in the cellular cysteine proteome are susceptible to oxidation (124). Cysteine, which is highly sensitive to redox response, widely distributed in a variety of regulatory sites such as cytoskeletons, enzymes, receptor membrane proteins, transcription factors, and nuclear proteins (125). Due to the rapid development of proteomics, the protein oxidation network of cysteine has been greatly supplemented. We attempt to shed light on the important biological effects of oxidative stress by briefly present several classic examples of oxidative signaling regulation.

**FIGURE 3:**
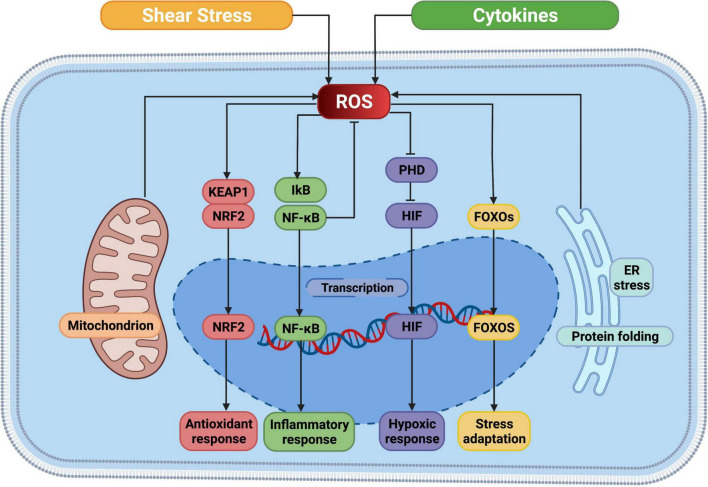
ROS mediated cellular signaling pathways. ROS, reactive oxygen species; NRF2, nuclear factor erythroid 2-related factor; KEAP1, Kelch-like ECH-associated protein 1; IkB, I-kappa B proteins; NF-κB, nuclear factor kappa B; PHD, prolyl hydroxylases; HIF, hypoxia-inducible factor; FOXO, forkhead box protein O; ER, endoplasmic reticulum. This figure was generated in Biorender (https://Biorender.com).

The Nrf2-Keap1 system is a thiol-based physiological sensor effect device that responds to oxidative stress and maintains redox homeostasis (126). Under normal physiological conditions, Keap1 binds tightly to Nrf2 in the cytoplasm and promotes Nrf2 ubiquitination and subsequent degradation processes. ROS acts on the multiple easily oxidizing cysteine residues contained in KEAP1 and causes conformational changes in KEAP1, thereby preventing NRF2 ubiquitination and promoting its nuclear transposition to initiate the expression of antioxidant defense proteins (127). In addition, the transcription factor nuclear factor-κB (NF-κB) is susceptible to redox regulation and controls inflammation as well as multiple biological processes like cell proliferation, cell death (128). On the one hand, intracellular ROS can regulate the oxidation and activation of NF-κB inhibitor (IκB) kinases to the negative NF-κB signaling pathway. On the other hand, due to the presence of oxidizable cysteine in the DNA binding region of NF-κB, ROS can directly activate the NF-κB signaling pathway (129). Hypoxia-inducible factor (HIF) is a major regulatory transcription factor in response to lower oxygen levels, including HIF-1, HIF-2, and HIF-3 (130). An important part of the cell response to hypoxia is ROS, which increases the stability of HIF-1. HIF prolyl hydroxylases (PHD), which sense oxygen availability and drive HIF hydroxylation, are inhibited by ROS both in hypoxia and normoxia (131). In turn, HIF-1 increases NOX expression, thereby promoting intracellular ROS production (132). The profound role of ROS is also reflected in the response to pressure sensors. For instance, the forkhead box protein O (FOXO) family is an important class of transcription factors that help maintain homeostasis in cells by integrating redox signaling. Similar to other regulatory modalities, direct oxidation of cysteine in members of the FOXO family leads to alter in downstream genes (133) (Figure 3).

### Preoperative inflammatory environment

Prior to the establishment of AVF, systemic changes associated with CKD lead to a surge in inflammation and oxidative stress in the circulatory system, which are present at all stages of AVF. The etiology of CKD is complex and is characterized by renal unit loss, decreased glomerular filtration rate, and irreversible changes in renal function and structure (134). On the one hand, elevated intracellular ROS levels cause the oxidation of lipids, DNA, and proteins, which plays an important role in CKD disease progression. On the other hand, oxidative stress levels in damaged kidneys can be further upregulated through mitochondrial energy metabolism and enzyme systems such as xanthine oxidase, peroxidase, nitric oxide synthase, and NADPH-oxidases (NOX) (135–137).

The production of ROS in mammalian cells mainly depends on organelles, including mitochondria and endoplasmic reticulum, and enzyme systems involving peroxidase, NADPH, and nitric oxide synthase (138). Mitochondria and NOX family oxidases are major contributors of ROS in kidneys (139, 140). Kidneys need a lot of energy to regulate the body’s water, electrolyte, and acid–base balance and for the maintenance of activities essential for life and normal operation. The reabsorption function of active transport in renal tubules is mainly driven by large amounts of adenosine triphosphate that are provided by mitochondria and double-membraned organelles (141). These organelles also regulate many cellular processes, such as cell proliferation, signaling, and cell death (142).

Therefore, mitochondrial dysfunction can have profound effects on kidney cell function. The essence of redox reactions is the gain or loss of electrons or the movement of shared electron pairs. Electrons are transferred between reducing agents and oxidants, and a stable reduction product is eventually formed. Mitochondria produce adenosine triphosphate through electron transfer via respiratory chain polymeric complexes in a process termed oxidative phosphorylation (142). In this process, the Nox4 subunit of NOX produces anionic superoxide via one-electron reduction of oxygen. The superoxide produced in the early stage is converted to H_2_O_2_ by the action of superoxide dismutase in the mitochondria and cytoplasm. In the oxidative phosphorylation chain, superoxide can be produced by oxygen electron loss in complexes I, II, and III (143, 144). Complex III produces ROS on both sides of the mitochondrial intima, whereas complexes I and II produce ROS only in the stroma (144). In addition, Nox2 increases mitochondrial ROS production through anti-electron transfer as a result of mitochondrial interactions with O_2_ derived from NOX (144). It has been reported that the impaired mitochondria can promote proinflammatory cytokines and chemokines through the release of ROS, cardiolipin and other harmful molecules, thereby inducing persistent kidney injury (145). Mitochondrial dysfunction in cisplatin-induced renal injury was due to inhibition of the activity of respiratory chain complexes I-IV, leading to ROS generation and a decrease in ATP production (146). Increased mitochondrial reactive oxygen species production has also been monitored in mice with diabetic nephropathy (147).

As CKD progresses, ROS levels increase, antioxidant levels decrease, and the oxidative balance is disrupted. Several studies have confirmed elevated levels of ROS in the blood of patients with CKD and animal models of kidney injury (148, 149). Antioxidants, including superoxide dismutase, glutathione, vitamin C, vitamin E, and the plasma sulfhydryl group, are significantly decreased due to overconsumption (150). In addition, nuclear factor erythroid 2-related factor (Nrf2), the upstream transcription factor of antioxidant products, was found to be downregulated in CKD patients (151).

### Intraoperative injury

Mechanical damage to the vein wall during surgery as well as pulling effects may cause local vessels to produce more ROS. Many studies have confirmed that ROS content in the outflow tract significantly increases in the short term after AVF surgery, and this process occurs prior to inward remodeling (152, 153). Alan Dardik and colleagues performed a gene chip microarray study on a mouse model of inferior vena cava and aorta shunting (7 days after surgery), demonstrating a high degree of early response regulation to injury after AVF. Microarray analysis showed that the different enriched genes are related to oxidative phosphorylation, mitochondrial long chain fatty acid β-oxidation, and mitochondrial unsaturated fatty acid. Beta-oxidation indicates that oxidative stress occurs in the early stage after AVF creation (154). In addition, cross-talk between factors of hypoxia and oxidative stress may play a more pronounced role in this process. In vascular anastomosis, the complete separation of long perivascular tissues is necessary. In addition to the transient interruption of blood flow during surgery, the rupture of trophoblast vessels in the venous wall leads to prolonged and irreversible hypoxia, which reduces the O_2_ concentration in the venous wall (155, 156). The venous vessels are in a low-flow and low-oxygen partial pressure environment for a long time before surgery (pO_2_ was about 35–45 mmHg). The establishment of an outflow canal can bring a large volume of blood to the vein, increasing the oxygen partial pressure up to levels in the arteries. It has been demonstrated that arterial oxygen levels stimulate NIH in veins through an ROS-dependent mechanism (157). All of the above conditions can cause oxidative stress through ischemia/reperfusion and stimulate the transmission of downstream signaling pathways.

### Postoperative hemodynamic changes

Arteriovenous fistula stenosis mainly occurs in the venous outflow tract near the anastomotic area. Local venous blood walls, in contrast to arteries, experience disrupted blood flow and can cause a range of gene expression changes and oxidative stress (135, 158). In an experimental study, WSS was shown to regulate several active species produced by the vessel wall under stable laminar flow conditions, keeping ECs and adjacent VSMCs in a relatively stable state. Continuous laminar flow of WSS within the normal physiological range and in the same stable direction activates downstream signaling pathways, inducing the expression of atheroprotective and antithrombotic genes with antioxidant, anti-inflammatory, anticoagulant, and antiapoptotic functions.

Kruppel-like factor 2 is one of the most important protective transcription factors regulated by WSS, and its main target is EC (159). The mechanism by which shear force increases KLF2 expression is associated with the activation of calmodulin-dependent kinase and subsequent phosphorylation of histone deacetylase 5 (160). Compared with steady state flow, pulsed fluid also upregulates KLF2 expression. KLF2 expression has been reported on the inner surface of thoracic aorta branches exposed to low WSS and not in outer surfaces exposed to high flow and high WSS (161).

Compared with unidirectional laminar flow, reciprocating WSS acts on EC to promote vascular atherosclerosis and increases the risk of thrombosis by enhancing the expression of pro-oxidative, pro-inflammatory, pro-coagulant, and other genes (162). KLF2 regulates endothelial function by regulating the eNOS/NO pathway. It has been confirmed that KLF2 upregulation in EC increases cell viability, decreases superoxide production of O_2_^–^ and ONOO^–^, and increases NO levels and eNOS activity. In addition, KLF2 can also promote eNOS uncoupling and regulate the production of antioxidant stress proteins by activating Nrf2/HO-1 (163).

In addition to influencing the release of NO and increasing the accumulation of ROS, a recent study suggested that WSS-induced inward remodeling is associated with heme HO (90). Studies have shown that heme-degrading enzymes are related to the patency of AVF (164). Heme degradation mainly includes two types: inducible HO-1 and constitutive HO-2. HO exerts vasodilatory, antioxidant, and anti-inflammatory effects through the production of carbon monoxide and biliverdin in addition to the release of ferrous iron from heme. Under the action of different modes of WSS, including high-intensity and low-intensity modes, the pathways and expression levels of HO-1 vary (89). HO-1 induced by high flow lies downstream of NO and mitochondrial-derived hydrogen peroxide. On the contrary, decreased HO-1 expression occurs in low-flow mode, which affects macrophage infiltration and superoxide production in the vascular wall. The result of a recent study in a mouse model of carotid artery injury suggests that HO-1 induced by NF-κB activation has vascular protective effects (164).

It was shown by the same study that HO-2 deficiency is associated with increased NIH. These *in vivo* studies suggest that the HO enzyme plays a fundamental role in promoting external remodeling and preventing NIH.

## Consequences of oxidative stress in arteriovenous fistula failure

### Oxidative stress and outward maturation

Outward maturation of AVF is a product of tube diameter enlargement and wall thickening and is considered to be an adaptive process that is triggered by sudden increases in blood pressure and shear stress and an environment with high partial pressure of oxygen. Vessel wall thickening is a process of vessel wall adaptation to pressure and is suitable for repeated puncture during subsequent dialysis. This process involves thickening all vascular layers through ECM deposition and cell proliferation and migration (165–167). A variety of cells are involved in vessel wall thickening, including VSMCs, adventitial fibroblasts, and bone marrow-derived progenitor cells (49, 55).

The outward remodeling of AVF is dependent on ECM-regulated synthesis, secretion, and degradation (168). Cells regulate ECM remodeling by degrading elastin and collagen through the secretory MMP family. ECM is a dynamic network structure existing between cells that is composed of macromolecular substances such as collagen, proteoglycans, and glycoproteins. These macromolecules can bind to specific receptors on the cell surface and alter gene expression through the direct binding of receptors connected to the cytoskeleton or triggering a signal transduction cascade in the cell, resulting in cell adhesion, migration, proliferation, and differentiation (169). Different tissues have different types and contents of ECM components. Among these, collagen mainly provides the basic framework and strength for tissue, and proteoglycan and hyaluronic acid are mainly involved in the maintenance of water levels and status and mechanical properties in addition to establishing and maintaining the concentration gradient of signaling molecules to ensure the development, form, and structure of tissue features (170).

Multiple studies show that oxidative stress plays a vital role in ECM metabolism and remodeling (171–173). The TGF-β1/Smad3 signaling pathway is one of the most important mechanisms regulating tissue fibrosis. ROS can promote ECM remodeling and fibrosis by activating the TGF-β1/Smad3 pathway, increasing connective tissue growth factor and MMP-2 expression (171). ROS can promote MMP-2 expression by activating the PI3-kinase/Akt signaling pathway, thereby activating bone marrow mesenchymal stem cells to synthesize ECM (172). Both Ras- and mitogen-activated protein kinase (MAPK)/ERK can activate extracellular regulated protein kinase (ERK). Activated ERK is translocated into the nucleus and participates in the regulation of transcription factor and cyclin expression, resulting in the proliferation and phenotypic changes in hematopoietic stem cells. Studies have shown that oxidative stress can inhibit the expression of insulin-like growth factor-1 by activating the MAPK/ERK signaling pathway and reducing ECM proteoglycan production in chondrocytes (174). In addition, ROS can upregulate TGF-β1 by activating the MAPK/ERK signaling pathway, stimulating the expression and secretion of MMP-9 in VSMCs, and regulating ECM metabolism (175). The Nrf2/ARE signaling pathway is an important response mechanism when the body suffers oxidative damage. In the state of oxidative stress, the activation of the Nrf2/antioxidant reactive element (ARE) pathway can inhibit the TGF-β/Smad signaling pathway and the decreased expression of collagen type I, fibronectin, tissue inhibitors of metalloproteinases (TIMPs), and PAI-1 (173).

During AVF outward remodeling, ECM synthesis, secretion, expression, and deposition occur at different time stages (176). Early ECM degradation after AVF formation coincides with an early increase in MMP and TIMP-1 expression. After 1 week, expression of many collagen subunits increases and MMP expression patterns are altered. Three weeks after surgery, later stages of remodeling are characterized by decreased expression of MMPs and increased expression of non-collagen matrix proteins (176). In AVF, the MMP family is the main factor regulating ECM degradation, with MMP-2 and MMP-9 upregulated in the early stage, and the increase in MMP-2 and TIMP in serum is an important indicator for predicting AVF maturation (68, 69). Conversely, matrix deposition is primarily regulated by TGF-β. TGF-β is produced by a variety of cells in the vessel wall, including endothelial, smooth muscle, and inflammatory cells, and can act by increasing ECM deposition, which significantly promotes thickening of the vessel wall (177).

### Oxidative stress and inward remodeling

**FIGURE 4:**
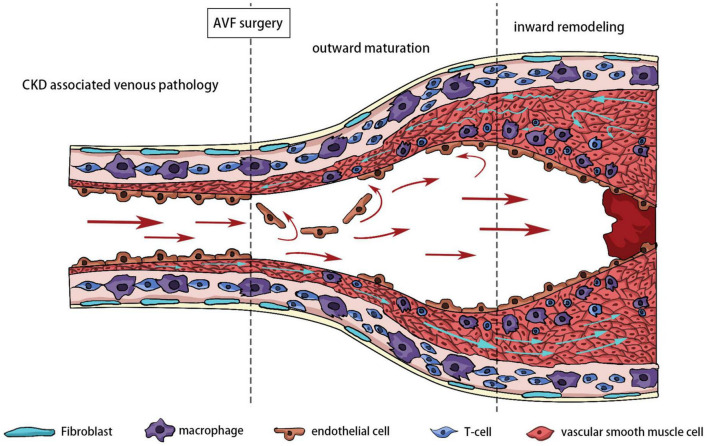
Different stages of outflow vein remodeling.

Inward remodeling of AVF refers to narrowing of the venous outflow tract due to aggressive intimal hyperplasia. The outflow vein is often stimulated by various factors, including hemodynamics, hypoxia, and oxidative stress at an early stage. The thickened venous wall is composed of matrix proteins and proliferating cells, called intimal hyperplasia. In the later stages after the establishment of AVF, the intimal hyperplasia is often not suppressed. Excessive intimal hyperplasia leads to stenosis of the lumen, and reduced blood flow renders the AVF unusable for dialysis. In extreme cases, stenosis can lead to complete occlusion of the blood vessel or even thrombosis. Pathological sectioning has shown that various cells, including ECs, VSMCs, adventitial fibroblasts, and inflammatory cells, are involved in NIH (49). The effects of oxidative stress on ECs, VSMCs, and fibroblasts have been extensively discussed (Figure 4).

The endothelium is the single layer of cells that coats the lining of the blood vessel lumen. ECs play a role in maintaining vascular homeostasis by regulating vascular tone and inflammatory responses and promoting angiogenesis. Endothelial activation, often the initial state of vascular injury, is characterized by the activation of inflammatory genes and downstream pathways. This ultimately leads to decreased NO production or bioavailability, impaired vasodilation, and other endothelial phenotypic changes collectively referred to as endothelial dysfunction.

**FIGURE 5:**
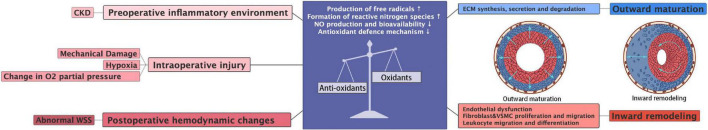
Source and consequences of imbalance oxidative stress in AVF.

The imbalance between ROS generation and the antioxidant defense system is one of the main causes of EC dysfunction, which is implicated in the development of various cardiovascular diseases such as atherosclerosis and thrombosis. Studies have shown that oxidative stress mediates EC activation by affecting the production and secretion of cytokines (178, 179) (Figure 5). In ECs, NO is a major factor in maintaining vascular homeostasis. The initial state of endothelial dysfunction involves a decrease in NO bioavailability. After oxidative stress, superoxide anions combine with NO to generate peroxynitrite ONOO^–^ (180). Peroxynitrite can promote protein nitration, leading to EC dysfunction and even death (181, 182). In the cardiovascular system, superoxide anion is often produced by enzymes such as NOX, xanthine oxidase, and unconjugated eNOS. Furthermore, the mitochondrial respiratory chain provides contributes significantly to the production of oxidative stress-related chemical substrates.

Following AVF creation, increased arterial flow directly leads to passive vasodilation and endothelial cell synthesis of NO (183, 184). NO is a gaseous small-molecule signal that is mainly produced by eNOS in ECs. Due to its anti-inflammatory, antithrombotic, and antiproliferative properties, NO plays an important role in the development of adaptive venous wall remodeling.

The expression of both eNOS and inducible nitric oxide synthase (iNOS) was significantly increased in the outflow tract of AVF, and the inhibition of eNOS results in increased inflammatory factors such as MCP-1 and IL-8, which exacerbate NIH (69). Endothelin-1 is an inflammatory mediator that promotes vasoconstriction and EC proliferation. Endothelin-1 expression was found to be upregulated in the vein wall, in the NIH region of AVF, and in the plasma of CKD and hemodialysis patients after endothelial activation (68). In addition, the expression of both p-selectin and e-selectin is upregulated early in AVF formation, promoting immune cell adhesion, and the expression of p-selectin decreases after 1 month. VCAM-1 is highly expressed in thrombosed and stenotic AVFs (71).

Typical sources of NIH proliferating cells are smooth muscle α-actin-positive VSMCs and fibroblasts. EC and VSMC injury, hemodynamic stress, and mechanical damage lead to the migration of VSMCs from the media to the intima and their differentiation into cells with a secretory phenotype (myofibroblasts). Fibroblast precursors in the adventitia of veins are able to sense sudden changes in the mechanical force generated by arterial flow, thereby rapidly adjusting their patterns of gene expression, secreting MMPs and collagen, and migrating to the intima. Cell proliferation is one of the important markers to measure the phenotypic transition of VSMCs (185). Numerous literature reports indicate that ROS promotes VSMC proliferation (186–188). In addition, ROS can also indirectly produce pro-proliferative effects on VSMC by mediating hormone and growth factor release. Studies have found that H_2_O_2_ can promote the proliferation of bradykinin, angiotensin-II, and growth factors such as PDGF and thrombin (189, 190). O_2_ mediates plasminogen urokinase-induced proliferation of VSMCs (191). Furthermore, ROS mediates bradykinin-induced VSMC proliferation and collagen production (192). In addition, the antioxidant N-acetylcysteine inhibits VEGF-induced VSMC proliferation and migration (193, 194). The nitroxide inhibitor Roebulin can attenuate the proliferation of VSMCs, indicating that NOXs play an important mediating role in the VSMC migration signaling pathway (195). The mechanism of PDGF-induced VSMC proliferation and migration has been widely discussed (196), and appears to mainly involve ROS binding and operate through PDGF-β receptors (197). α and β receptors are almost always expressed in VSMCs (198). PDGF-mediated migration signaling cascades are known to include MAPKs ERK1/2, Jun-N-terminal kinase (JNK), and p38 (199).

A number of growth factors are upregulated after AVF creation, including VEGF, PDGF, basic fibroblast growth factor, and insulin-like growth factor-1, among others (11, 156, 200). These processes are closely linked with oxidative stress generation. In animal experiments using p47phox (subunits of NADPH) knockout AVF rats, it was found that decreasing the oxidative stress production can result in reductions in the proliferation of smooth muscle cells in the lumen and secretion of inflammatory factors and improve the patency rate (201). One week after AVF production, the expression of adhesion molecule β-catenin and proto-oncogene c-Myc increases due to the decrease in levels of adhesion molecule N-cadherin, which is linked to VSMC proliferation (202). In addition, local oxidative stress can also be alleviated by inhibiting the Notch signaling pathway, which results in decreased migration and proliferation of smooth muscle cells (54).

## Oxidative stress in endovascular interventions treating arteriovenous fistula failure

### Endovascular intervention for arteriovenous fistula failure

Arteriovenous fistula has been recognized as the primary and optimal hemodialysis vascular access option for ESRD patients requiring renal replacement therapy (203–205). Despite being widespread, there are undeniable limitations associated with the use of AVFs in hemodialysis. In a 2008 clinical trial, investigators found that more than 60% of fistulas did not mature, and failure to mature means they are defined as unfit for dialysis (6). The vast majority of arteriovenous fistulas fail due to immaturity or NIH. Traditional surgical revision is the most direct way to treat dialysis access stenosis. However, percutaneous transluminal angioplasty is the current standard for the treatment of arteriovenous stenosis due to its reduced invasiveness and the convenience of multiple treatments (206, 207). The principle of all endoluminal repair of AVF is to expand the lumen and increase the volume, mainly including balloon-assisted maturation (BAM), stent placement, and cutting balloon angioplasty.

The BAM technique uses balloon angioplasty to repeatedly inflate the vein wall to dilate the diameter of the fistula to facilitate smooth dialysis (208, 209). The most common complication after balloon dilation remains restenosis due to postoperative intimal hyperplasia, and although this is more common in arteries, veins may respond similarly to angioplasty, especially in the inflammatory setting of renal failure.

Although evidence from long-term large data volumes is lacking, several short-term studies have shown positive results. One report mentioned that 85% of 53 patients experienced secondary patency within 1 year of BAM (208). Miller et al. found that of 122 immature fistulas using BAM, dialysis after treatment was possible in 118 cases, with a secondary patency rate of 75% after 1 year (210). Gallagher and colleagues performed 185 BAMs on 45 patients (an average of 3.7 BAMs per patient), resulting in 37 patients with usable dialysis access (211).

Stenting is another tool for the management of refractory dialysis access strictures. Multiple studies have reported substantial improvements in primary and secondary patency rates for fistulas and grafts after stenting. A prospective multicenter randomized trial testing polytetrafluoroethylene-covered self-expanding nitinol stents showed higher patency rates in the stent-graft group than in the balloon angioplasty alone group after 6 months (212). A meta-analysis of 10 studies showed that after 6 months, patients who received nitinol stents had improved initial patency rates compared with those who received angioplasty (213).

Cutting balloon angioplasty, wherein a balloon with several small blades is axially mounted on the outer surface of the cutting balloon, is used to dilate the stenosis, which causes trauma to the vessel wall similarly to BAM. It can be used for balloon expansion while cutting the proliferative part of the vascular disease site (214). Several studies have reported significant improvements in vascular patency after 6 months of use (215–217).

### Oxidative stress in endovascular interventions

Oxidative stress largely affects the pathogenesis and outcome of various cardiovascular diseases. Endovascular interventions, including balloon angioplasty and stenting, are associated with increased levels of ROS in the vessel wall and altered endothelial and smooth muscle cell function. These changes lead to restenosis and thrombosis of blood vessels.

Several studies have discussed the mechanism of ROS generation by endovascular intervention. The mechanism of ROS generated by interventional therapy is largely similar, and they all lead to the accumulation of ROS after damage to ECs, vascular elastic lamina, and vascular media through mechanical stress or damage. Shortly after injury, O_2_^–^ levels in the vessel wall are elevated and colocalize with VSMCs (218, 219). NOX-mediated O_2_^–^ production was observed in the adventitial layer of vessels, revealing an important potential role of fibroblasts in ROS production (220). In addition, the researchers found that the expressions of Nox1, Nox4, gp91phox, and p22phox were significantly upregulated after carotid balloon injury in rats (218). Balloon angioplasty can rapidly induce p38 mitogen-activated protein kinase, which is also activated by ROS and is involved in VSMC hypertrophy and NIH (221). Mechanistic studies on the increased oxidative stress after stent placement have also been reported. The expression of two NOX subunits, p22phox and gp91phox, was increased after stenting in the rabbit carotid artery, resulting in higher ROS production (222).

In addition, ROS resulting from interventional therapy can affect the state of smooth muscle and the diastolic function of blood vessels through endothelial dysfunction. The severity of endothelial disorder tends to be closely related to the type of surgery. Compared with balloon angioplasty, stent placement tends to result in more severe endothelial damage (223). Endothelial dysfunction is a proinflammatory and procoagulant state of ECs after various physicochemical stimuli, characterized by altered expression of adhesion molecules on the EC surface and the recruitment of various immune cells (224). Several studies have shown that oxidative stress plays a key role in mediating endothelial dysfunction (85, 178, 179). In ECs, NO is a major factor in the maintenance of vascular homeostasis. The initial stage of endothelial dysfunction is accompanied by reduced NO production, increased dissociative consumption, or reduced bioavailability. Superoxide anion usually reacts with NO to form peroxynitrite ONOO (225, 226). Peroxynitrite, in turn, promotes protein nitration, leading to EC dysfunction and death (182, 227).

## Oxidative stress as a therapeutic target in arteriovenous fistula failure

The disruption of oxidative homeostasis is the result of excess production of ROS not counteracted by the intrinsic antioxidant defense system, which largely contributes to endothelial dysfunction, cellular proliferation, and AVF failure. Targeting oxidative stress for the treatment of AVF failure has been extensively studied in preclinical settings and has shown highly promising results in clinical studies, which are summarized in Tables 1, 2. These studies all achieved similar results in increasing patency rates and reducing intimal hyperplasia, focusing on three different approaches to target oxidative stress in AVF failure: (1) inhibiting endogenous oxidative stress production and (2) increasing endogenous antioxidant capacity, both directly, or indirectly via (3) supplementing exogenous antioxidants.

**TABLE 1:** Antioxidant therapies in AVF failure.

Mechanism	Target	Treatment	Model	Results	References
Inhibition of the source of reactive oxygen species (ROS)	Nuclear factor kappa B	Pyrrolidine dithiocarbamate administration	Human umbilical vein endothelial cells, arteriovenous fistulas (AVF) mice	Blocked ROS production *in vitro*, slowed vessel remodeling	(236)
	Monoamine oxidases	Incubation with clorgyline or selegiline	Fragments of brachial artery collaterals harvested from end-stage renal disease patients	Reduced ROS level and improved maturation and long-term patency of fistula	(237)
	NADPH oxidase	p47phox knockout	AVF mice	Reduced ROS and delayed the vessel remodeling process	(201)
	NADPH oxidase HMG-CoA reductase	Rosuvastatin administration	AVF rats with diabetes mellitus	Improved luminal dilation and blood flow by suppressing the levels of superoxide anions and proinflammatory activities	(238)
				Improved blood flow and endothelial function by attenuating activity of proinflammatory genes and generation of superoxide anions	(239)
Improving endogenous antioxidant capacity	Superoxide dismutase, heme oxygenase-1, and catalase	Protandim medium addition	Human saphenous veins cultured *ex vivo*	Reduced levels of superoxide, blocked intimal hyperplasia and reduced cellular proliferation	(240)
	Heme oxygenase-1 (HO-1)	HO-1 gene knockout	AVF mice	Easier restenosis, accelerated neointimal hyperplasia, and increased vasculopathic gene expression in HO-1^–/–^ AVF mice	(90)
Supplementation of exogenous antioxidants	ROS	Tempol administration	AVF rats	Decreased neointima formation in the juxta-anastomotic venous segment and improved AVF blood flow	(153)

**TABLE 2:** Antioxidant therapies in AVF failure.

Mechanism	Target	Treatment	Patients (*n*)	Trial ID/testing status	Results	References
Inhibition of ROS producers	NADPH oxidase HMG-CoA reductase	Atorvastatin p.o. 40 mg/d for 3 days	Undergoing coronary artery bypass grafting (21)	NCT01013103, completed	Reduced O_2_^–^ generation in saphenous vein grafts used for coronary artery bypass grafting surgery	(241)
		Rosuvastatin p.o. 5 mg once daily for 4 weeks	Arteriovenous fistula in diabetic patients with chronic renal failure (30)	NCT01863914, completed		(238)
	Lysyl oxidase	D-Penicillamine (25 μM) in combination with ascorbic acid (10.0 μM)	Arteriovenous fistula occlusion (10)	NCT03106948, recruiting		
Improving endogenous antioxidant capacity	Heme oxygenase-1	Determination of length polymorphism of (GT)n repeats in HO-1 gene promoter	Hemodialysis (HO-1 genotyping: S/S: 148, S/L: 297, L/L: 158)	Completed	Longer length polymorphism of (GT)n repeats in the HO-1 gene was correlated with a higher frequency of pathway failure and poorer AVF patency	(232)
		Determination of length polymorphism of (GT)n repeats in HO-1 gene promoter and far-infrared therapy	Hemodialysis (HO-1 genotyping: S/S: 55, S/L: 116, L/L: 68)	ACTRN12610000704099, completed	The lowest incidence of AVF malfunction in S/S group	(242)
Supplementation of exogenous antioxidants	ROS	Ascorbic acid i.v. 300 mg, 600 mg × 3 week for 3 months	Post-angioplasty of hemodialysis (31)	NCT03524846, completed	Might attenuate restenosis after angioplasty	(243)
		Coenzyme Q10 dose-escalation p.o. 300, 600, 1200 and 1800 mg daily, each for 14 days	Chronic hemodialysis (15)	NCT00908297, completed	Improved mitochondrial function and decreased oxidative stress	(244)
		α-Lipoic acid administration 600 mg/d	Diabetic patients on hemodialysis (30)	Completed	Showed anti-inflammatory and antioxidant activity, improved anemia, controlled blood glucose and reduced cardiovascular risk	(245)
		Combination of tocopherols (666 IU/d) plus α-lipoic acid (600 mg/d) over 6 months	Maintenance hemodialysis therapy (160)	NCT00237718, completed	Safe and well tolerated but no effect on biomarkers of inflammation and oxidative stress or the erythropoietic response	(246)
		Vitamin C i.v. plus a vitamin E-coated dialyzer	Chronic hemodialysis with anemia and atherosclerosis (20)	Completed	Palliated oxidative stress such as hemolysis and lipid peroxidation	(247)
		Combination of atorvastatin (once daily 40 mg) and a-tocopherol (once daily 800 IU)	End-stage renal disease with maintenance hemodialysis therapy (11)	Completed	Decreased plasma total cholesterol, triglycerides, low-density lipoprotein (LDL), apoB and oxLDL and had good impacts on *in vitro* LDL oxidizability	(248)
		Folic acid 10 mg supplementation	Uremia with arteriovenous fistula failure (100)	ChiCTR-IPR-17013111, not yet recruiting		
		Coenzyme Q10 400 mg	Maintenance hemodialysis therapy over 3 months (30)	ChiCTR1900022258, not yet recruiting		

The information was obtained from clinicaltrials.gov.

The initial discussion of AVF came from the discovery of increased oxidative stress and growth factor expression following AVF failure in human specimens (11). The study found that the oxidative stress marker 4-hydroxy-2,3-nonenol colocalized with TGF-β, a growth factor known to promote intimal hyperplasia. For the first time, the potential of oxidative stress as a therapeutic target in dialysis access failure was revealed. Subsequent experimental animal studies focused on the effects of oxidative stress on AVF by uncoupling NOX or NOS. There are many important ROS-generating systems in the blood vessel wall, among which NOX is one of the most important. NOX triggers eNOS uncoupling, elevates xanthine oxidase activity, and enhances mitochondrial ROS production (228–230). Stephanie Lehoux and colleagues were the first to report that neointima is suppressed and AVF patency is improved in mice lacking the NOX p47phox (201). In addition, they also explored the effects of endogenous ROS and NO on the activity and expression of MMPs. The generation of ROS through NOS uncoupling has also been investigated as a possible target for addressing AVF failure. Tsapenko and colleagues reported an increase in the production of peroxynitrite, a product of superoxide anion interaction with nitrogen oxide (153). Under pathological conditions, NOS activity is uncoupled, resulting in increased superoxide anion production. In fact, NOS decoupling is closely related to vascular diseases such as heart failure and atherosclerosis. To test the evidence for this uncoupling, the authors demonstrated that the ratio of tetrahydrobiopterin to dihydrobiopterin, a reliable marker for NOS uncoupling, was significantly reduced in the venous segment of the AVF. These findings suggest that the overproduction of targeted ROS has a profound impact on failure to treat AVF.

In addition to suppressing oxidative production, some experimental studies have shown that increasing the endogenous antioxidant capacity can improve AVF patency. The findings of Nath and colleagues detail the role of endogenous antioxidant stress kinases in AVF dysfunction. After AVF creation, an increase in the expression of the antioxidant enzyme HO-1 is induced due to an increase in superoxide anion (153). HO-1 catalyzes the decomposition of heme into equal amounts of iron, carbon monoxide, and biliverdin (231). Biliverin reductase activity further reduces biliverdin to bilirubin. Both bile pigments can scavenge peroxyl free radicals, while carbon monoxide can cause vasodilation. Thus, induction of HO-1, as an adaptive response to injury, is critical for protection from AVF dysfunction. Earlier, a study by Juncos et al. showed that HO-1 production could be induced in a mouse model of AVF (90). In HO-1^–/–^ mice, AVF exhibited lower patency rates. Furthermore, longer GT repeats are shown to be closely associated with reduced HO-1 expression and AVF pathway failure in patients with HO-1 gene polymorphisms, again highlighting the important role of oxidative stress in AVF (232).

Following these inspiring results from therapeutic studies involving increased endogenous antioxidant enzymes, several experimental studies attempted to explore the effects of exogenous antioxidant supplementation on AVF. Tempol is a widely used free radical scavenger with powerful antioxidant properties. Nath and colleagues reported that the use of tempol significantly improved the AVF patency and permeability and, at high doses, also increased blood flow to the AVF (153).

## Conclusion

There is increasing evidence that multiple upstream and downstream effects at different time periods in AVF maturation trigger increased oxidative stress and mediate intimal hyperplasia. At present, excellent preliminary results have been achieved in animal experiments for the treatment of AVF stenosis against oxidative stress. Preclinical studies have also shown promising results through various antioxidant strategies; however, these beneficial effects have unfortunately not been translated into positive outcomes for advancing clinical treatment. The main reason for this is that the antioxidative stress drugs used in clinical research often lack specificity. In addition, the systemic modalities previously tested for AVFs often fail to produce sufficient local antioxidative stress effects. We expect more periadventitial drug delivery systems to show favorable prospects in AVF treatment.

The role of oxidative stress in AVF formation may appear to be a double-edged sword, but its positive effects are often overlooked. On the one hand, much evidence points to the negative effects of oxidative stress on AVF maturation by causing endothelial dysfunction and stimulating the proliferation and migration of smooth muscle cells and fibroblasts. On the other hand, the massive production of oxidative stress in the early stage of AVF formation also plays an undeniably important role in AVF outward remodeling and maturation. Perhaps the research on AVF should go deeper into the change law of oxidative stress in the whole process to understand the changing role of oxidative stress generation in different stages from maturation to inward remodeling of AVF. Likewise, research should delve into the outcomes of modulating antioxidant stress at different time points.

## Author contributions

WW and KH contributed to the conceptualization. KH, YG, YXL, CL, CC, SZ, and ZK contributed to the investigation. KH and YG contributed to the writing—original draft preparation. WW, YQL, SZ, ZK, CL, and YXL contributed to the writing—review and editing. WW and YQL contributed to the supervision. All authors have read and agreed to the published version of the manuscript.
